# Totally Endoscopic Coronary Artery Bypass Graft: Systematic Review and Meta-Analysis of Reconstructed Patient-Level Data

**DOI:** 10.1177/15569845241296530

**Published:** 2024-11-20

**Authors:** Ioannis Zoupas, Vasiliki Manaki, Panagiotis T. Tasoudis, Nina-Rafailia Karela, Dimitrios V. Avgerinos, Konstantinos S. Mylonas

**Affiliations:** 1 Cardiothoracic and Vascular Surgery Working Group, Society of Junior Doctors, Athens, Greece; 2 Department of Cardiac Surgery, Hygeia Hospital, Athens, Greece; 3 Department of Vascular Surgery, AHEPA University Hospital, Thessaloniki, Greece; 4 Department of Neurosurgery, “Panagiotis & Aglaia Kyriakou” Children’s Hospital, Athens, Greece; 5 Department of Cardiac Surgery, Onassis Cardiac Surgery Center, Athens, Greece; 6 Department of Pediatric Cardiothoracic Surgery, University of Rochester, NY, USA

**Keywords:** coronary artery bypass graft, CABG, endoscopic, robotic, cardiac surgery, minimally invasive

## Abstract

**Objective:**

The standard approach for coronary artery bypass grafting is open surgery. Totally endoscopic coronary artery bypass has emerged as an alternative for selected patients. This meta-analysis sought to evaluate clinical outcomes with this emerging technique.

**Methods:**

A PRISMA-compliant search was performed up to December 14, 2022, in PubMed (MEDLINE), Scopus, and Cochrane. Time-to-event data were reconstructed using Kaplan–Meier curves from source literature.

**Results:**

A total of 2,774 patients with symptomatic coronary artery disease underwent totally endoscopic coronary artery bypass in 18 eligible studies. The mean patient age was 63.2 ± 12.3 years, and 77.5% (95% confidence interval [CI]: 72.2% to 82.4%) of the included patients were males. The mean operative time was 304.2 ± 155 min, whereas the mean internal mammary artery takedown time was 38.3 ± 18.4 min. Of the patients, 4.7% (95% CI: 1.6% to 9.1%) required conversions to open surgery. The 30-day complication rate was 5.9% (95% CI: 1.2% to 13.1%), whereas late complications developed in 4.8% (95% CI: 1.9% to 8.5%) of the patients. Freedom from major adverse cardiac events was 93.4% (95% CI: 85.3% to 94.8%) and 1-year, 5-year, and 10-year survival rates were 95.2%, 83.2%, and 81.7%, respectively. Reintervention was required in 3.3% (95% CI: 2.3% to 4.4%) of the cohort within a mean follow-up of 42.5 ± 27.8 months.

**Conclusions:**

Totally endoscopic coronary artery bypass may be a safe and viable alternative for selected patients with coronary artery disease. Long-term follow-up will help define the place of robotic endoscopic treatment in the armamentarium of myocardial revascularization.

## Graphical abstract

**Visual abstract:**
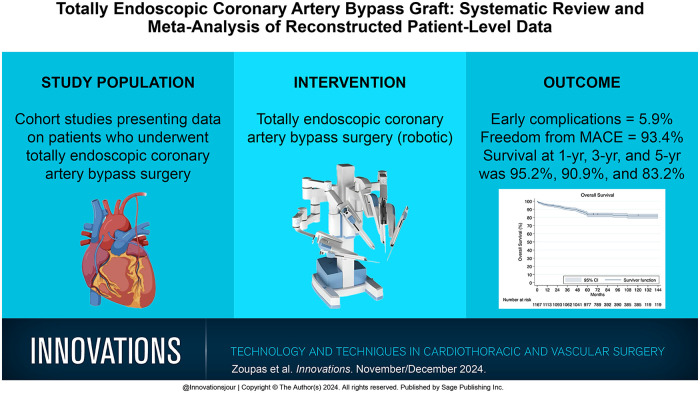

**Central Message**

The results of this review of the literature found that totally endoscopic coronary artery bypass provides satisfactory short-term and long-term outcomes for selected patients, constituting a viable alternative to the well-established open technique.

## Introduction

Robotic totally endoscopic coronary artery bypass (TECAB) was first performed in 1998 by Dr Didier Loulmet and his team at Broussais Hospital in Paris, France.^1^ The number of cardiac procedures performed either entirely or partially robotically grew after the Food and Drug Administration approved the da Vinci robotic surgical system (Intuitive Surgical, Sunnyvale, CA, USA) for cardiac surgery in 2002.^2^ In the past 2 decades, advances in technology have enabled single-vessel to multivessel endoscopic graft implantation with or without cardiopulmonary bypass (CPB). Fourth-generation robotic systems are now available, and specialized robotic equipment for coronary bypass surgery has been developed.^3^

Expert endoscopic cardiac surgery groups have reported reduced postoperative morbidity, length of hospital stay, and overall costs with totally endoscopic coronary surgery compared with its conventional counterpart.^4^ Not surprisingly, most published experience with TECAB stems from single-institution analyses.^5–15^ Previous systematic reviews have failed to define the impact of TECAB on perioperative metrics including morbidity, need for reintervention, and patient survival.^2,3,16–21^

To summarize data from existing literature research on TECAB, we conducted a systematic review and meta-analysis of individual patient data (IPD) based on prospective cohort, nonrandomized propensity score–matched, and retrospective cohort studies.

## Methods

### Systematic Search and Eligibility Criteria

Our study was conducted in accordance with the Preferred Reporting Items for Systematic Reviews and Meta-Analyses (PRISMA) statement (Supplemental Fig. 1) and the protocol that was agreed upon by all authors. Included studies were published in English and reported original patient data (randomized controlled trials, nonrandomized prospective clinical studies, retrospective clinical studies, and case series). Only studies reporting adult patients undergoing TECAB were considered eligible. Reviews, meta-analyses, case reports, irrelevant studies, papers with insufficient patient data, editorials, letters to the editor, comments, personal opinions, errata, and manuscripts written in languages other than English were disqualified.

A systematic search was conducted up to December 14, 2022, and assessed studies from PubMed/MEDLINE, Scopus, and the Cochrane database. Two independent reviewers (I.Z. and V.M.) searched these registries using the following algorithm: ((totally) AND (endoscopic)) AND (“coronary artery bypass” OR (CABG) OR “bypass surgery”). All conflicts regarding study eligibility were resolved with the assistance of a third, more experienced reviewer (P.T.T.). At the beginning, a protocol of our work was submitted to PROSPERO and was registered with the following ID number: CRD42023392947.

### Data Extraction

Two independent reviewers performed data extraction using a standardized predesigned formula for evidence collection. Conflicts were brought to a third researcher’s attention and resolved. The following patient features were extracted: age, sex, body mass index, left ventricular ejection fraction, EuroSCORE II, Society of Thoracic Surgeons score, smoking habits, presence of diabetes mellitus, coronary artery disease (CAD), peripheral artery disease, chronic obstructive pulmonary disease (COPD), hypertension, connective tissue disease, dyslipidemia, prior percutaneous coronary intervention (PCI), prior arrhythmia, prior myocardial infarction (MI), prior cardiothoracic surgery, cardiovascular disease, kidney disease, dialysis history, and heart failure.

Operation time (min), single or multiple grafts, internal mammary artery (IMA) takedown, use of intra-aortic balloon pump (IABP), bleeding, cross-clamp time (min), CPB time (min), type of robotic platform used, concomitant procedures, intensive care unit stay (days), hospital stay (days), postoperative ventilation time (min), graft failure, and follow-up (months), were also extracted.

Finally, we reviewed information regarding the presence of postoperative arrhythmias, postoperative renal failure, postoperative cerebrovascular accident (CVA), postoperative MI, respiratory and other complications, reoperation rates, postoperative PCI, cardiac arrest, as well as mortality rates (30-day and overall), cardiac death and other-cause death, freedom from major adverse cardiac events (MACE), freedom from angina, and conversion to open surgery. In case of missing data regarding specific parameters, we only extracted data available in each study.

### Statistical Analysis

#### Data pooling and patient feature meta-analysis

The Hozo et al. and Wan et al. techniques were used to calculate the standard deviations and mean values of continuous variables whenever medians and ranges or median and interquartile ranges were provided.^22,23^ To assess the occurrence rates of several events and the presence of between-study heterogeneity, we used the χ²-based Q statistic (significant if *P* < 0.10) and *I*^2^ value. All analyses were performed using STATA MP 17.0 (StataCorp LLC, College Station, TX, USA).

#### Reconstruction of patient time-to-event data and survival meta-analysis

We reconstructed IPD from Kaplan–Meier (KM) curves of the 18 included studies, whenever they were available. We followed the methodology described by Wei and Royston.^24^ High-quality screenshots from the KM curves were acquired, and Web Plot Digitizer was used to digitize them. Every added spot had to represent a specific combination between time points and their corresponding survival information, so that this information could be extracted. Each of the added spots represented a patient of the included study. Isotonic regression was used to identify deviations from monotonicity, which were managed with a pool-adjacent-violators algorithm. Extractable IPD were then incorporated into STATA MP 17.0 to create a new KM curve that would include data from all available KM curves. This method provided us with pooled data regarding survival rate.

#### Risk-of-bias assessment

The quality of the data presented in the included studies was evaluated using the National Heart, Lung, and Blood Institute scale (Supplemental Table). The follow-up time sufficiency cutoff value was set at 12 months after the operation for each study.

## Results

### Literature Search

The search initially yielded a total of 633 records. Ultimately, 18 studies met all inclusion criteria and were used for data collection.^4–12,14,15,20,25–32^ There were 16 retrospective observational studies and 2 prospective ones, with 1 randomized clinical trial. Following tabulation, we analyzed data from 2,774 adult patients undergoing TECAB (Table 1).

**Table 1.** Study Characteristics of the Included Manuscripts.

Author	Year	Country	Design	Study period	TECAB, *n*
Argenziano et al.	2006	United States, Austria	RCT	2002–2004	85
Balkhy et al.	2021	United States	Retrospective cohort	2013–2020	544
Balkhy et al.	2011	United States	Retrospective cohort	2008–2010	120
Cheng et al.	2021	China	Retrospective cohort	2007–2017	126
Claessens et al.	2022	Belgium	Retrospective cohort	2016–2018	244
Dhawan et al.	2012	United States	Retrospective cohort	2007–2009	106
deCanniere et al.	2007	Belgium, Germany	Retrospective cohort	1998–2002	228
Dogan et al.	2002	Germany	Retrospective cohort	1999–2002	62
Efendiev et al.	2015	Russia	Prospective cohort	2012–2015	50
Jegaden et al.	2011	France	Retrospective cohort	1998–2008	59
Kappert et al.	2008	Germany	Retrospective cohort	1999–2001	41
Mishra et al.	2006	India	Retrospective cohort	2002–2005	13
Mohr et al.	2001	Germany	Retrospective cohort	2000	27
Pasrija et al.	2018	United States	Retrospective cohort	2011–2014	50
Srivastava et al.	2012	United States	Retrospective cohort	2008–2009	164
Srivastava et al.	2010	United States	Retrospective cohort	2004–2007	214
Wiedemann et al.	2013	United States, Austria	Retrospective cohort	2001–2011	500
Zaouter et al.	2015	France	Retrospective cohort	2011–2014	38

Abbreviations: RCT, randomized controlled trial; TECAB, totally endoscopic coronary artery bypass.

### Basic Demographics and Medical History

Overall, 77.5% (95% confidence interval [CI]: 72.2% to 82.4%) of the patients were male, and the mean age at the time of the operation was 63.2 ± 12.3 years. In terms of additional comorbidities, 38.5% (95% CI: 31.3% to 46.0%) were active smokers, 70.3% (95% CI: 57.9% to 81.4%) had dyslipidemia, 4.4% (95% CI: 0.6% to 10.9%) had history of chronic kidney disease, 28.8% (95% CI: 22.6% to 35.4%) had diabetes mellitus, and 8.1% (95% CI: 1.2% to 20.0%) had heart failure. In addition, 6.9% of the patients (95% CI: 3.9% to 10.5%) had peripheral arterial disease, 6.5% (95% CI: 3.2% to 10.7%) had COPD, 72.8% (95% CI: 65.3% to 79.8%) presented with history of hypertension, and 8.3% (95% CI: 6.4% to 10.5%) suffered from cerebrovascular disease. Connective tissue disorders were not reported by any of the included studies. In addition, 2.6% of the patients (95% CI: 1.7% to 3.5%) had undergone prior cardiothoracic surgical procedures, whereas 25.5% (95% CI: 18.4% to 33.4%) had prior PCI performed. Finally, the mean EuroSCORE II for the included patients was 3.15 ± 3.2. Significant details regarding demographics and prior medical history are described in Table 2.

**Table 2.** Details of Patient Demographic Characteristics and Medical History.

Author	TECAB, *n*	Age, years	Male	Female	DM	COPD	HTN
Argenziano et al. (2006)	85	58.4 (9.6)	69 (81.2)	16 (18.8)	18 (21.9)	NR	47 (57.3)
Balkhy et al. (2021)	544	66 (10.5)	411 (76)	133 (24)	219 (40)	52 (9.6)	451 (83)
Balkhy et al. (2011)	120	66.3 (10.4)	86 (72)	34 (28)	23 (19.4)	15 (12.5)	73 (61.1)
Cheng et al. (2021)	126	59.1 (9)	105 (83.3)	21 (16.7)	37 (29.4)	NR	73 (57.9)
Claessens et al. (2022)	347	66.9 (10.1)	295 (85.01)	52 (14.99)	114 (32.9)	NR	225 (64.84)
Dhawan et al. (2012)	106	63.6 (11.5)	79 (74.5)	27 (25.5)	36 (34)	NR	98 (92.5)
deCanniere et al. (2007)	228	59.2 (10.1)	NR	NR	NR	NR	NR
Dogan et al. (2002)	62	NR	NR	NR	13 (20.3)	0	NR
Efendiev et al. (2015)	50	NR	NR	NR	NR	NR	NR
Jegaden et al. (2011)	59	59 (12)	53 (89.8)	6 (10.2)	NR	NR	NR
Kappert et al. (2008)	41	60.6 (8.9)	36 (88)	5 (12)	12 (30)	NR	33 (82)
Mishra et al. (2006)	13	56.3 (7.2)	12 (92.3)	1 (7.7)	5 (38.5)	1 (7.7)	6 (46.2)
Mohr et al. (2001)	27	62 (8)	20 (74)	7 (26)	NR	NR	NR
Pasrija et al. (2018)	50	64.77 (5.67)	30 (60)	20 (40)	26 (52)	NA	43 (86)
Srivastava et al. (2012)	164	63 (11)	128 (78)	36 (22)	9 (17.7)	2 (1.2)	91 (55.5)
Srivastava et al. (2010)	214	67.9 (11.8)	111 (52)	103 (48)	79 (37)	11 (5.1)	180 (84.1)
Wiedemann et al. (2013)	500	59 (18.54)	364 (72.8)	136 (27.2)	134 (26.8)	55 (11)	407 (81.4)
Zaouter et al. (2015)	38	64 (10)	33 (86.8)	5 (13.2)	14 (37)	7 (18)	28 (74)

Abbreviations: DM, diabetes mellitus; COPD, chronic obstructive pulmonary disease; HTN, hypertension; NR, not reported; TECAB, totally endoscopic coronary artery bypass.

Data are reported as mean (SD) or *n* (%).

### Disease and Perioperative Details

The operation was performed with the use of a da Vinci telemanipulation system in all cases. Overall, 85.0% (95% CI: 74.2% to 93.4%; *n* = 1,628) of the patients underwent single-vessel coronary artery bypass grafting (CABG) procedures, of which 97.9% were for LIMA to left anterior descending artery (LAD). Moreover, 13.7% (95% CI: 5.8% to 24.2%; *n* = 670) of the cohort underwent double-vessel CABG, 1.6% (95% CI: 0.5% to 3.1%; *n* = 81) had triple-vessel CABG, whereas only 4 patients underwent quadruple-vessel CABG. Furthermore, both IMAs were used in 527 patients (7.8%, 95% CI: 1.5% to 17.8%), and 24.0% (95% CI: 5.2% to 50.4%) of operations were performed on-pump versus 76.0% (95% CI: 51.4% to 94.0%) off-pump. Only 2% of the cohort (95% CI: 0.9% to 3.4%) experienced technical failure during the installation of the graft.

The mean operation time was 304.2 ± 155 min, whereas the mean IMA takedown, CPB, cross-clamping, and postoperative ventilation times, when performed, were 38.3 ± 18.4 min, 133.5 ± 95.9 min, 71.3 ± 51.4 min, and 71.1 ± 136.9 h, respectively. IABP was introduced in 1.4% (95% CI: 0.7% to 2.2%) of the cohort. Details about the number of bypassed vessels and use of the heart–lung machine are reported in Table 3.

**Table 3.** Number of Bypassed Vessels and Cardiopulmonary Bypass Usage.

Author	TECAB, *n*	Single-vessel CABG	Double-vessel CABG	Triple-vessel CABG	On-pump CABG	Off-pump CABG
Argenziano et al. (2006)	85	85 (100)	0	0	85 (100)	0
Balkhy et al. (2021)	544	238 (44)	265 (49)	40 (7)	0	544 (100)
Balkhy et al. (2011)	120	78 (65.0)	37 (30.8)	5 (4.16)	0	120 (100)
Cheng et al. (2021)	126	105 (83.3)	NR	NR	0	126 (100)
Claessens et al. (2022)	347	NR	NR	NR	347 (100)	0
Dhawan et al. (2012)	106	30 (28)	67 (63)	8 (7.5)	3 (2.8)	103 (97.2)
deCanniere et al. (2007)	228	205 (90)	NR	NR	117 (51.3)	111 (48.7)
Dogan et al. (2002)	62	50 (80.6)	12 (19.4)	0	54 (87)	8 (13)
Efendiev et al. (2015)	50	50 (100)	0	0	0	50 (100)
Jegaden et al. (2011)	59	56 (95)	3 (5)	0	0	59 (100)
Kappert et al. (2008)	41	37 (90.2)	4 (9.8)	0	8 (19.6)	33 (80.4)
Mishra et al. (2006)	13	13 (100)	0	0	2 (15.4)	11 (84.6)
Mohr et al. (2001)	27	27 (100)	0	0	27 (100)	0
Pasrija et al. (2018)	50	50 (100)	0	0	28 (56)	22 (44)
Srivastava et al. (2012)	164	93 (56.7)	64 (39)	6 (3.7)	0	164 (100)
Srivastava et al. (2010)	214	139 (65)	68 (31.8)	7 (3.3)	0	214
Wiedemann et al. (2013)	500	334 (66.8)	150 (30)	15 (3)	98 (19.6)	402 (80.4)
Zaouter et al. (2015)	38	38 (100)	0	0	0	38 (100)

Abbreviations: CABG, coronary artery bypass graft; NR, not reported; TECAB, totally endoscopic coronary artery bypass.

Data are reported as *n* (%).

In addition, 11.2% (95% CI: 5.1% to 19.1%) underwent a hybrid procedure with PCI, whereas 6.4% (95% CI: 0% to 21.0%) of the patients underwent other concomitant procedures during TECAB. In total, 4.7% (95% CI: 1.6% to 9.1%; *n* = 198) required conversion to open surgery. Overall, LIMA–LAD patency in the study cohort was achieved in 652 patients (96.1%, 95% CI: 93.3% to 98.3%), with a total of 683 patients available for angiographic follow-up.

Based on available data, 51 patients (41.8%) required conversion to sternotomy, and 71 (58.2%) underwent minithoracotomy. Etiologies for conversion to another approach were available for 127 patients. Eleven patients (8.7%) had unsuitable LAD (small diameter or extreme calcification), 2 (1.6%) developed ventricular arrhythmia intraoperatively, 5 (3.9%) suffered injury to the IMA or the left ventricle, 16 (12.6%) had new-onset hemodynamic instability, 10 (7.9%) had graft failure, 2 (1.6%) developed myocardial ischemia, 22 (17.3%) had bleeding from the anastomosis site, 6 (4.7%) had CPB time that exceeded the acceptable value, and 52 (40.9%) were converted due to miscellaneous undefined surgical technical difficulties.

The mean length of stay in the intensive care unit was 1.9 ± 2.1 days. The average length of hospital stay was 5.4 ± 4.2 days. The 30-day complication rate was 5.9% (95% CI: 1.2% to 13.1%), whereas 4.8% (95% CI: 1.9% to 8.5%) developed late complications (>30 days after the operation). Overall, 23 patients (0.7%, 95% CI: 0.1% to 1.9%) experienced postoperative bleeding, 19 (0.4%, 95% CI: 0.01% to 1.1%) had postoperative MI, 21 (0.9%, 95% CI: 0.4% to 1.7%) had postoperative stroke, 9 (0.1%, 95% CI: 0% to 0.7%) had postoperative renal failure, and 32 (1.6%, 95% CI: 0.2% to 3.9%) had postoperative respiratory complications, whereas 104 (1.1%, 95% CI: 0% to 6.6%) experienced postoperative atrial fibrillation and 23 (0.5%, 95% CI: 0% to 1.6%) had cardiac arrest after the surgery. Finally, 1 patient experienced cannulation-induced femoral artery retrograde dissection. Details about early and late morbidity including conversion to open surgery are provided in Table 4.

**Table 4.** Cumulative Data About Complications and Conversion to Open Surgery Rates.

Author	TECAB, *n*	Early complications	Early complication types	Late complications	Late complication types	Conversion to open surgery
Argenziano et al. (2006)	85	1	Graft failure (*n* = 1, 1.2%)	NR	NA	5 (5.9)
Balkhy et al. (2021)	544	164	AF (*n* = 71, 13%) AKI (*n* = 16, 2.9%) CVA (*n* = 1, 0.2%) MI (*n* = 1, 0.2%) Bleeding (*n* = 6, 1.1%)	12	MI (*n* = 12, 2.2%)	1 (0.2)
Balkhy et al. (2011)	120	11	MI (*n* = 1, 0.8%) CVA (*n* = 1, 0.8%) Bleeding (*n* = 2, 1.6%) Respiratory failure (*n* = 2, 1.6%) Pericardial effusion (*n* = 1, 0.8%) Pleural effusion (*n* = 2, 1.6%)	5	Graft failure (*n* = 5, 4.1%)	3 (2.5)
Cheng et al. (2021)	126	2	Graft failure (*n* = 1, 0.7%) Bleeding (*n* = 1, 0.7%)	6	CVA (*n* = 3, 2.3%) Graft failure (*n* = 3, 2.3%)	1 (0.8)
Claessens et al. (2022)	347	13	Cardiac death (*n* = 5, 1.4%) MI (*n* = 2, 0.6%) CVA (*n* = 3, 0.8%)	44	Cardiac death (*n* = 12, 3.4%) MI (*n* = 9, 2.6%) CVA (*n* = 9, 2.6%)	8 (2.3)
Dhawan et al. (2012)	106	26	AKI (*n* = 8, 7.5%) CVA (*n* = 2, 1.9%)	NR	NA	12 (11.3)
deCanniere et al. (2007)	228	NA	MI (*n* = 2, 0.9%)	NR	NA	64 (28)
Dogan et al. (2002)	62	NA	Bleeding (*n* = 4, 6.4%) Graft failure (*n* = 2, 3.2%) CVA (*n* = 1, 1.6%) AF (*n* = 3, 4.8%)	NR	NA	16 (24.2)
Efendiev et al. (2015)	50	0	NA	0	NA	0
Jegaden et al. (2011)	59	7	Bleeding (*n* = 5, 8.5%) MI (*n* = 2, 3.3%)	10	Recurrent angina (*n* = 8, 13.5%) Graft failure (*n* = 2, 3.3%)	0
Kappert et al. (2008)	41	0	NA	2	MI (*n* = 2, 4.9%)	0
Mishra et al. (2006)	13	1	Bleeding (*n* = 1, 7.7%)	1	Graft failure (*n* = 1, 7.7%)	0
Mohr et al. (2001)	27	0	NA	2	Graft failure (*n* = 1, 3.7%) Respiratory failure (*n* = 1, 3.7%)	6 (22.2)
Pasrija et al. (2018)	50	1	Respiratory failure (*n* = 1, 2%)	NR	NA	2 (4)
Srivastava et al. (2012)	164	2	CVA (*n* = 1, 0.6%) Cardiac death (*n* = 1, 0.6%)	4	RCA dissection (*n* = 1, 0.6%) Graft failure (*n* = 3, 1.8%)	0
Srivastava et al. (2010)	214	NR	NR	NR	NA	17 (7.9)
Wiedemann et al. (2013)	500	NR	NR	NR	NA	63 (12.6)
Zaouter et al. (2015)	38	11	AF (*n* = 7, 18.4%)	NR	NA	1 (2.6)

Abbreviations: AF, atrial fibrillation; AKI, acute kidney injury; CVA, cerebrovascular accident; MI, myocardial infarction; NA, not applicable; NR, not reported; RCA, right coronary artery; TECAB, totally endoscopic coronary artery bypass.

Data are reported as *n* (%).

### Reinterventions

**Fig. 1.**
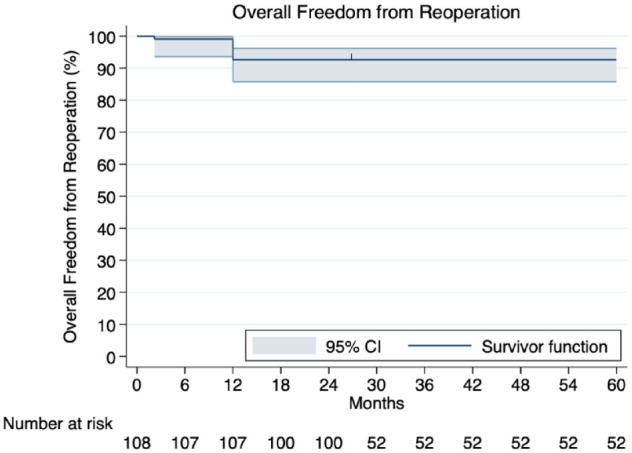
Kaplan–Meier curve demonstrating overall freedom from reintervention constructed by individualized patient data. CI, confidence interval.

Reinterventions were required in 3.3% (95% CI: 2.3% to 4.4%) of patients within a mean follow-up time of 42.5 ± 27.8 months. Information about the type of reintervention was available for 58 patients. Specifically, PCI was performed in 28 patients and redo surgical repair in 30 patients. Perioperative findings are summarized in Table 5, and a reconstructed KM curve regarding overall freedom from reintervention is depicted in Figure 1. A diagram regarding reintervention rates is shown in Supplemental Figure 2.

**Table 5.** Perioperative Details.

Author	TECAB, *n*	Operation time, min	IMA takedown time, min	Follow-up time, months	Concomitant procedures, *n*	Mean hospital stay, days	Mean ICU stay, days
Argenziano et al. (2006)	85	353 (89)	60 (24)	NR	0	5.1 (3.4)	1.46 (1.54)
Balkhy et al. (2021)	544	251 (84)	NR	38 (NR)	52	2.72 (1.29)	1.3 (0.69)
Balkhy et al. (2011)	120	286 (121)	NR	7.2 (2.2)	4	3.3 (2.4)	NR
Cheng et al. (2021)	126	184.7 (36.5)	24.3 (9.5)	NR	NR	NR	1.09 (0.44)
Claessens et al. (2022)	347	NR	NR	NR	103	8.9 (7.6)	3.55 (3.69)
Dhawan et al. (2012)	106	451 (142)	NR	NA	NR	5.2 (3.1)	2.3 (2.5)
deCanniere et al. (2007)	228	NR	NR	6 (0)	NR	NR	NR
Dogan et al. (2002)	62	280.6 (62.6)	64.3 (19.6)	NR	NR	9.2 (3.5)	1.54 (3.12)
Efendiev et al. (2015)	50	133 (47)	20 (17)	12 (0)	0	NR	0.54 (0.12)
Jegaden et al. (2011)	59	204 (42)	NR	21.6 (1.2)	NR	5.5 (1.6)	0.96 (0.8)
Kappert et al. (2008)	41	NR	NR	69 (7.4)	NR	NR	NR
Mishra et al. (2006)	13	236 (45)	48.7 (13.3)	24 (NR)	NR	4.5 (NR)	1.2 (NR)
Mohr et al. (2001)	27	348 (96)	64 (64)	3 (NR)	NR	9.4 (2.9)	0.6 (0.3)
Pasrija et al. (2018)	50	206.9 (17.9)	NA	1 (NR)	20	8.01 (1.52)	2.17 (0.89)
Srivastava et al. (2012)	164	254.6 (63)	34.1 (9.6)	6 (NR)	29	NR	NR
Srivastava et al. (2010)	214	233.5 (71.3)	34.1 (11.8)	17.6 (23.2)	50	NR	NR
Wiedemann et al. (2013)	500	444.8 (282.9)	NR	29 (NR)	219	17 (16.4)	11.5 (13.9)
Zaouter et al. (2015)	38	NR	NR	NA	1	8 (1.2)	1.1 (0.3)

Abbreviations: ICU, intensive care unit; IMA, internal mammary artery; NR, not reported; TECAB, totally endoscopic coronary artery bypass.

Data are reported as mean (SD) or *n* (%).

### Overall Survival and Mortality

**Fig. 2.**
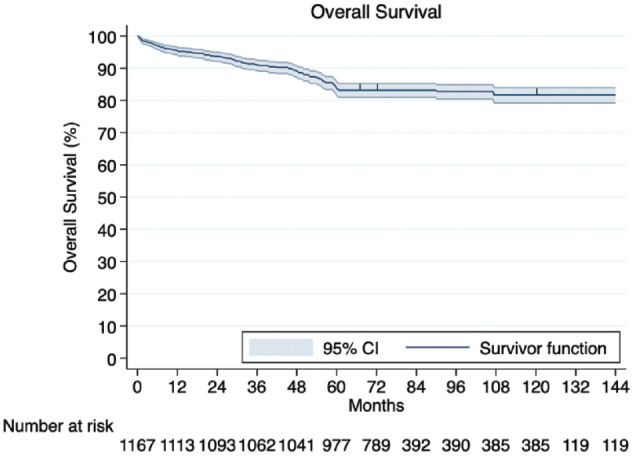
Kaplan–Meier curve demonstrating overall survival constructed by individualized patient data. CI, confidence interval.

The pooled 30-day mortality rate was 0.4% (95% CI: 0.1% to 0.7%). Overall mortality was estimated at 1.8% (95% CI: 0.2% to 4.5%), with cardiac-related death accounting for 31.1% (95% CI: 5.2% to 63.3%), whereas other causes of death were found in 68.9% (95% CI: 36.7% to 94.8%) of the fatalities. Forest plots demonstrating pooled event rates for mortality are provided in Supplemental Figure 3 and Supplemental Figure 4. Based on the IPD, 1-year, 5-year, and 10-year survival rates following TECAB were 95.1%, 83.2%, and 81.7%, respectively (Fig. 2).

### Freedom from Major Adverse Cardiac and Cerebrovascular Events

**Fig. 3.**
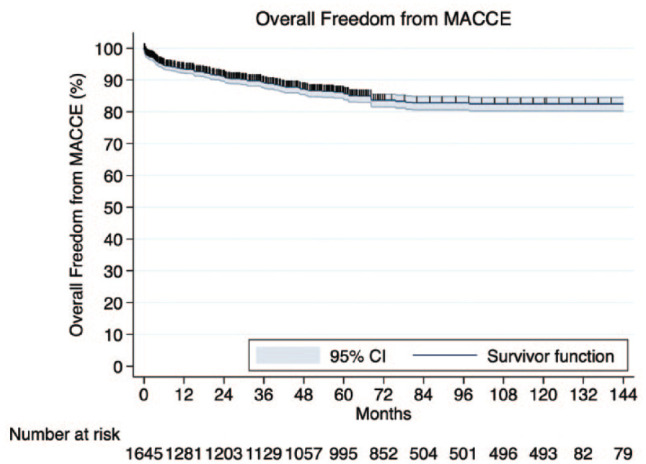
Kaplan–Meier curve demonstrating overall freedom from MACCE survival constructed by individualized patient data. CI, confidence interval; MACCE, major adverse cardiac and cerebrovascular events.

Freedom from MACE (reintervention, MI, and mortality) was calculated at 93.4% (95% CI: 85.3% to 94.8%). According to the IPD, 1-year, 5-year, and 10-year freedom from major adverse cardiac and cerebrovascular events (MACCE) was 93.4%, 85.8%, and 82.3%, respectively (Fig. 3). Finally, in 91.9% (95% CI: 79.5% to 99.1%) of the patients, no recurrent angina was observed during follow-up.

## Discussion

The potential benefits of TECAB include reduced surgical trauma, shorter length of hospital stay, earlier return to daily activities, minimalization of pain, and overall better quality of life compared with the conventional open approach. TECAB has been proven to be safe and reproducible by multiple cardiovascular surgical centers worldwide. However, widespread adoption of the procedure still faces significant challenges, including expensive specialized equipment with additional costs, significant technical difficulty, a challenging learning curve, prolonged operating time, and single-lung ventilation.^17,33,34^

The present meta-analysis includes 18 studies with a total of 2,774 patients, making it the most thorough and mathematically robust summary of CAD management with TECAB to date. In our study, the mean length of hospital stay was less than 6 days with a 30-day mortality rate of 0.36%. On the other hand, landmark studies such as the SYNTAX (1.3%) and FREEDOM trials (1.7%) reported markedly higher early mortality after CABG.^32,35,36^ This decrease might be explained by the fact that patients chosen for TECAB usually suffer from less severe comorbidities and are subject to more strict selection criteria than those undergoing conventional CABG. In our study, 76% of patients received operations on a beating heart, with about 85% of the procedures being single-vessel TECAB. Notably, the reintervention rate was 3.31% within a mean follow-up of 42.5 months. In relation to the most recent meta-analysis by Leonard et al.,^2^ what we add to the literature is mathematical robustness by applying the individual patient-level data technique and the incorporation of more recent studies since 2018. To date, this is the most mathematically comprehensive meta-analysis for TECAB providing the most robust and applicable results.

For our study, the overall rate of conversion to sternotomy or minithoracotomy was 4.69%. The most common reasons for conversions were various surgical technical difficulties and bleeding from the anastomotic site. Conversion rates, technical difficulties, operative times, and complications tend to decrease when the learning curve is reached, and team training has been achieved.^16,33^ In general, for robotic cardiac procedures, the steep part of this curve is surpassed after 30 to 50 repetitions by a single surgeon, whereas significant improvements in the results are observed when a triple-digit number is reached. Of all robotic cardiac operations, TECAB is considered the most demanding, requiring a much different and more complex skill set from its open counterpart. Multidisciplinary team training, careful institutional assembly, and single-surgeon experience with both minimally invasive and open cardiac procedures are some of the minimal requirements for the success and maintenance of an endoscopic coronary surgery program and the accomplishment of its full therapeutic benefit.^17,34,37^

Although TECAB has been established for more than 2 decades, many centers moved away from it after an initial period of enthusiasm. This shift largely resulted from technical challenges and the widespread adoption of other safe, well-established alternatives. In 2016, Rodriguez et al.^37^ had proposed team training—highlighting the role of a surgeon-team leader and the value of experience from numerous operations and varied patient scenarios—for the entire realm of endoscopic cardiac surgery. Three years later, after conducting the first 2 TECAB cases in New York state, Torregrossa and Puskas suggested methods to enhance the efficacy of TECAB in both clinical practice and surgical education.^38^ Key recommendations included the introduction of advanced simulators for surgeon training, the creation of new robotic tools and instruments specifically for the harvesting and skeletonization of the IMA, and the use of a refined distal anastomotic connector (Flex-A anastomotic connector, Cardica, Inc., Redwood, CA, USA) to further innovate and simplify the procedure. Bonatti et al. echoed the significance of developing distal anastomotic connectors for TECAB in 2019.^17^ They also emphasized that team training, mock surgeries, a step-by-step approach, and 3-dimensional vision for all surgical team members could greatly enhance surgical outcomes and elevate training quality for this particular technique. It is evident that there is substantial potential for enhancement, and if these modifications are adopted, TECAB could see broader acceptance.

The rate of postoperative complications is a pivotal factor in our analysis. Of the patients studied, 5.9% experienced complications within the first 30 days after surgery, whereas 4.8% faced complications subsequently. Notably, the prevalent complications were respiratory-related (1.6%), atrial fibrillation (1.1%), and CVA (0.9%). As per the SYNTAX and FREEDOM trials as well as additional literature,^32,36,39^ the rates of postoperative stroke and arrhythmias (primarily atrial fibrillation) are heightened following CABG in comparison with TECAB. Yet, Kofler et al., in their study on TECAB, articulated that the long-term outcomes between conventional CABG and TECAB patients are relatively comparable. They noted that the conventional CABG group exhibited significantly greater preoperative risk factors. This might be due to surgeons opting for the tried-and-true CABG method that they are familiar with, reserving the innovative TECAB approach for more stable and lower-risk patients. Their data, focusing on unadjusted groups, revealed that perioperative MACCE rates were comparable for both techniques, whereas the robotic group had a marginally lower perioperative mortality.^32^ This aligns with our aggregate analysis. In our study, early mortality after surgery was recorded at a mere 0.4%, contrasting with figures from the SYNTAX trial. Moreover, MACCE was observed in roughly 10% of our study’s patients, whereas a 5-year follow-up of the SYNTAX trial reported a 24.2% MACCE rate among CABG patients. Finally, with conventional CABG providing superb outcomes regarding LIMA–LAD patency rates, it is of great importance to note that TECAB does not fall short with a measured patency rate of more than 96%. These observations significantly underscore the safety, efficacy, and potential superiority of TECAB when executed by a proficient team.

Although our results are promising, it is important to understand that not every CAD surgical case is suitable for TECAB. Bonatti et al. outlined an exhaustive list of both absolute and relative contraindications for TECAB. These include limited space within the left pleural cavity, internal adhesions, an enlarged heart situated close to the chest wall, acute myocardial ischemia, pronounced pulmonary dysfunction, extreme obesity, and severe multiorgan dysfunction. All these factors can lead to the operation’s failure and necessitate a switch to an open procedure, while also introducing supplementary risks to survival and the quality of the anastomosis.^17^ Additional contraindications include diminutive or extensively diseased and calcified target vessels that could compromise the anastomosis quality. Other factors, including narrow femoral arteries, moderate to acute aorto-iliac atherosclerosis, an ascending aortic diameter exceeding 35 mm, and blockages in the inferior vena cava or iliac vein, render the operation unfeasible due to challenges in CPB access and cardioplegia delivery. Swiftly identifying these issues and promptly transitioning to conventional methods can ensure a satisfactory surgical outcome and reduce unexpected complications.^17^

Methodological strengths of the present article include (1) comprehensive literature search using a rigorous and systematic methodology, (2) detailed patient-level extraction of time-to-event data, (3) standardized quality assessment of eligible studies, and (4) construction of KM statistics using IPD. The main differences between our study and the older meta-analysis^3^ are (1) the addition of 377 more patients, (2) the extensive overlap check of the studies since TECAB is performed only in specific centers worldwide, and (3) the reconstruction of IPD using KM curves. Regarding differences in inclusion criteria, we also chose to include adult patients operated for CAD both on arrested and on beating heart for either single-vessel or multivessel CABG.

The main limitations of this systematic review and meta-analysis are the retrospective observational design of most included publications with lack of randomization leading to an unavoidable degree of selection bias. Furthermore, the limited adoption of TECAB for treatment of CAD has led to the publication of various experiences with variable learning curves in different stages of progress and training. This may jeopardize the generalizability and applicability of our results, as we could not stratify them in terms of experience. Lastly, eligible studies did not provide granular data regarding their open surgical cohorts. Therefore, we could not perform a direct comparison of open surgery versus TECAB.

## Conclusions

The present study used robust patient-level meta-analysis techniques to summarize contemporary experience with TECAB grafting. This is the first and only meta-analysis on TECAB using this mathematically robust statistical method. Conversion occurred in less than 5% of the cohort and 30-day mortality did not exceed 0.4%. The incidence of postoperative stroke, MI, and atrial fibrillation was also remarkably low. The 1-year, 3-year, and 5-year survival rates were estimated at 95.2%, 90.9%, and 83.2%, respectively. Reintervention was required in less than 4% of the patients over a mean follow-up of 42.5 months. These encouraging findings suggest that TECAB might be a viable option for selected low-risk patients. Further research with longer follow-up and randomization is warranted to further define the role of robotic surgery in the setting of CABG.

## Supplemental Material

sj-pdf-1-inv-10.1177_15569845241296530 – Supplemental material for Totally Endoscopic Coronary Artery Bypass Graft: Systematic Review and Meta-Analysis of Reconstructed Patient-Level Data Supplemental material, sj-pdf-1-inv-10.1177_15569845241296530 for Totally Endoscopic Coronary Artery Bypass Graft: Systematic Review and Meta-Analysis of Reconstructed Patient-Level Data by Ioannis Zoupas, Vasiliki Manaki, Panagiotis T. Tasoudis, Nina-Rafailia Karela, Dimitrios V. Avgerinos and Konstantinos S. Mylonas in Innovations
