# Supplementary material for: Totally Endoscopic Coronary Artery Bypass Graft: Systematic Review and Meta-Analysis of Reconstructed Patient-Level Data
Source: Innovations (Phila). 2024 Nov 20;19(6):616–25. doi: 10.1177/15569845241296530 (PMC11656624; doi:10.1177/15569845241296530)

Supplemental Table. Quality Assessment of Included Case Series Using the NHLBI Scale.

Author	Study question or objective clearly stated?	Study population clearly and fully described, including a case definition?	Were the cases consecutive?	Were the subjects comparable?	Was the intervention clearly described?	Outcomes measured clearly defined, valid, reliable, and implemented consistently across all study participants?	Length of follow-up adequate?	Were the statistical methods well-described?	Were the results well-described?
Argenziano et al. (2006)	YES	YES	YES	YES	YES	YES	NO	YES	YES
Balkhy et al. (2021)	YES	YES	YES	YES	YES	YES	YES	YES	YES
Balkhy et al. (2011)	YES	YES	YES	YES	YES	YES	NO	NR	YES
Cheng et al. (2021)	YES	YES	YES	YES	YES	YES	YES	YES	YES
Claessens et al. (2022)	YES	YES	YES	YES	YES	YES	YES	YES	YES
Dhawan et al. (2012)	YES	YES	YES	YES	YES	YES	NO	YES	YES
deCanniere et al. (2007)	YES	YES	YES	YES	YES	YES	NO	YES	YES
Dogan et al. (2002)	YES	YES	YES	YES	YES	YES	NR	NR	YES
Efendiev et al. (2015)	YES	YES	YES	YES	YES	YES	YES	NR	YES
Jegaden et al. (2011)	YES	YES	NO	NR	YES	YES	YES	YES	YES
Kappert et al. (2008)	YES	YES	NO	YES	YES	YES	YES	YES	YES
Mishra et al. (2006)	YES	YES	YES	YES	YES	YES	YES	YES	YES
Mohr et al. (2001)	YES	YES	YES	YES	YES	YES	YES	YES	YES
Pasrija et al. (2018)	YES	YES	YES	YES	YES	YES	NO	YES	YES
Srivastava et al. (2012)	YES	YES	YES	YES	YES	YES	NO	YES	YES
Srivastava et al. (2010)	YES	YES	YES	YES	YES	YES	YES	YES	YES
Wiedemann et al. (2013)	YES	YES	YES	YES	YES	YES	YES	YES	YES
Zaouter et al. (2015)	YES	YES	YES	YES	YES	YES	NO	YES	YES

Abbreviation: NHLBI, National Heart, Lung, and Blood Institute; NR, not reported.

Supplemental Figure 1. Flow diagram in accordance with the PRISMA 2020 guidelines.

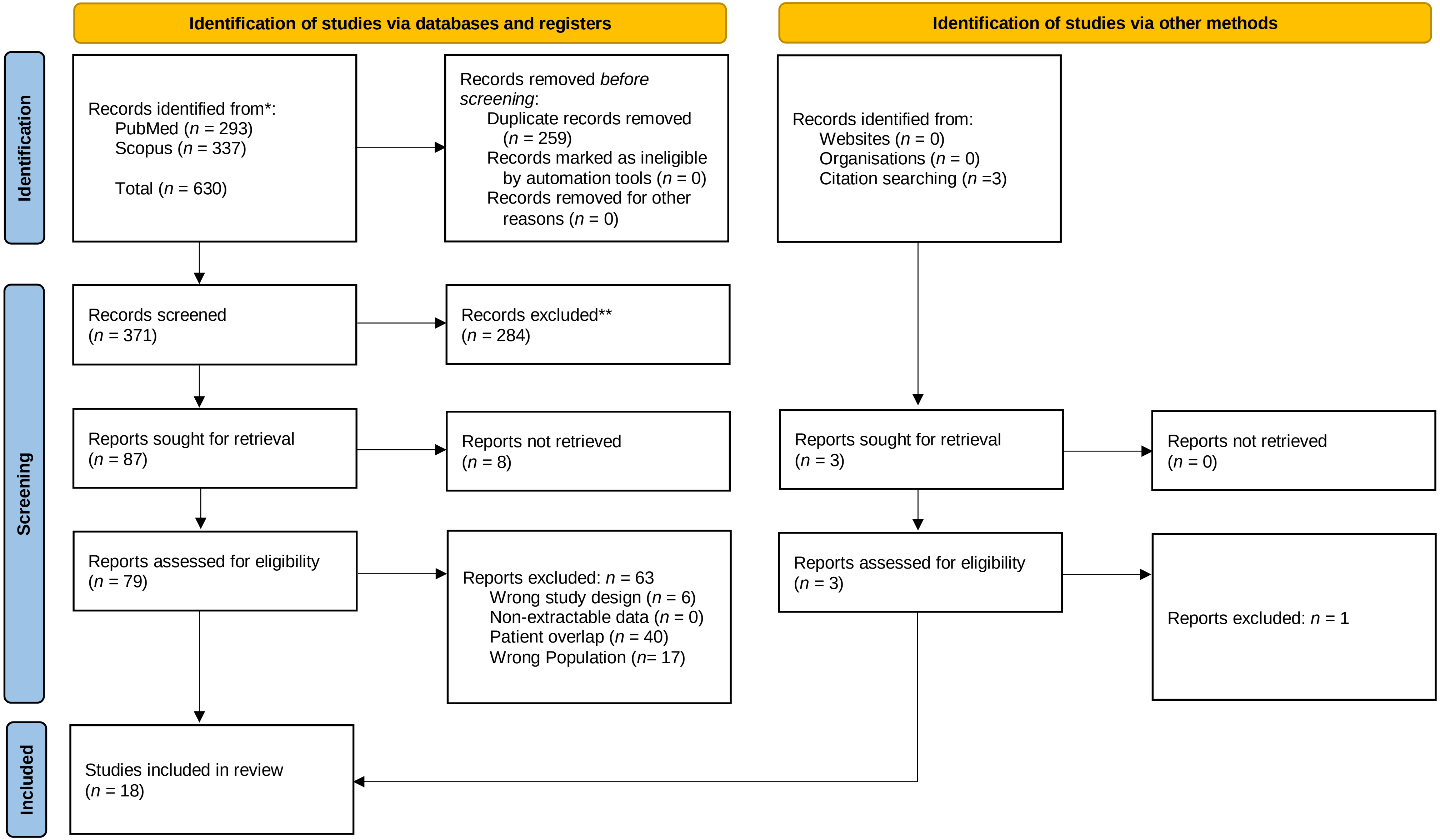

*Consider, if feasible to do so, reporting the number of records identified from each database or register searched (rather than the total number across all databases/registers).
**If automation tools were used, indicate how many records were excluded by a human and how many were excluded by automation tools.

From: Page MJ, McKenzie JE, Bossuyt PM, Boutron I, Hoffmann TC, Mulrow CD, et al. The PRISMA 2020 statement: an updated guideline for reporting systematic reviews. BMJ 2021;372:n71. doi: 10.1136/bmj.n71. For more information, visit: http://www.prisma-statement.org/

Supplemental Figure 2. Forest plot diagram demonstrating data for reintervention rates.

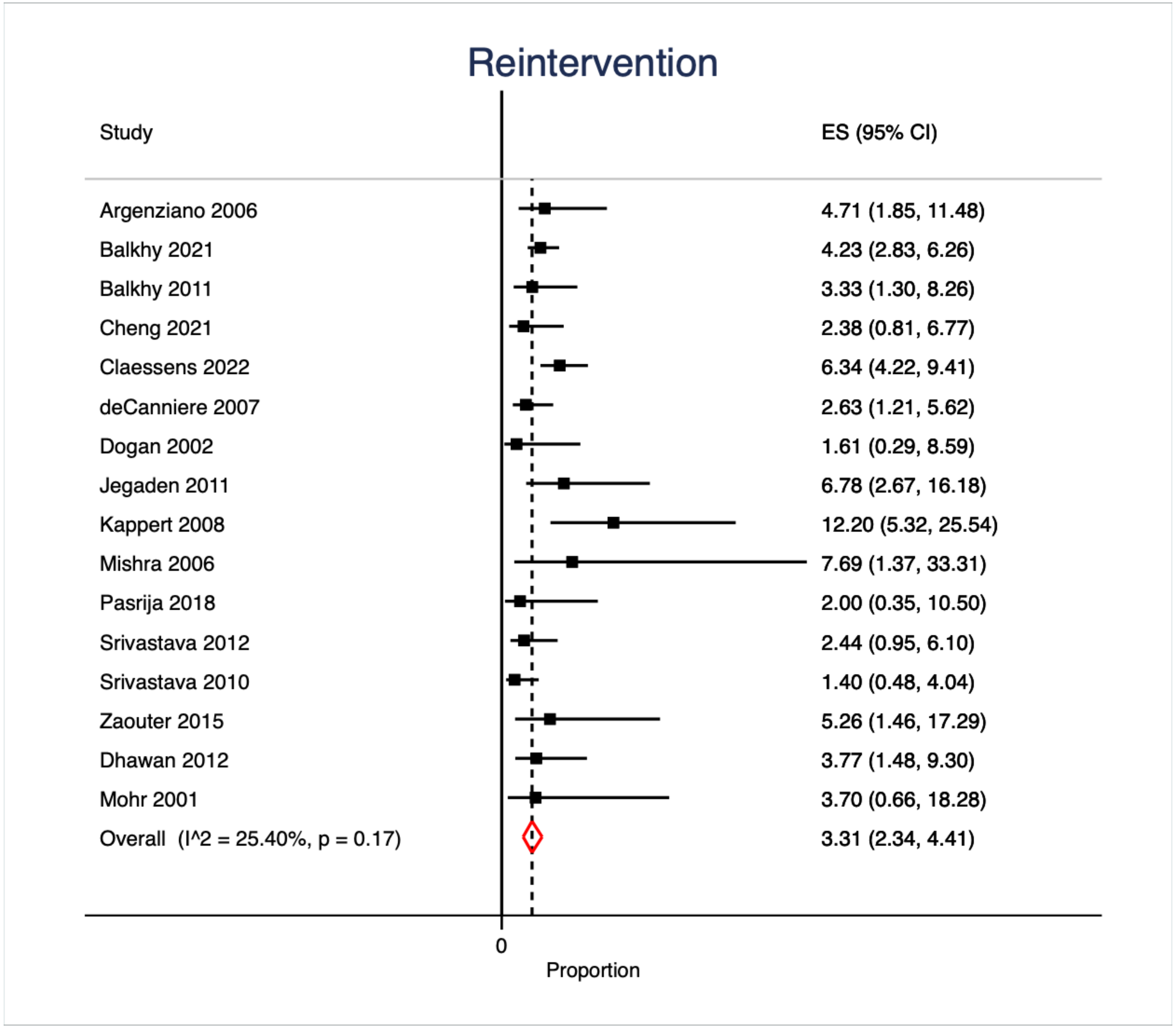

Supplemental Figure 3. Forest plot diagram demonstrating data for early mortality event rates.

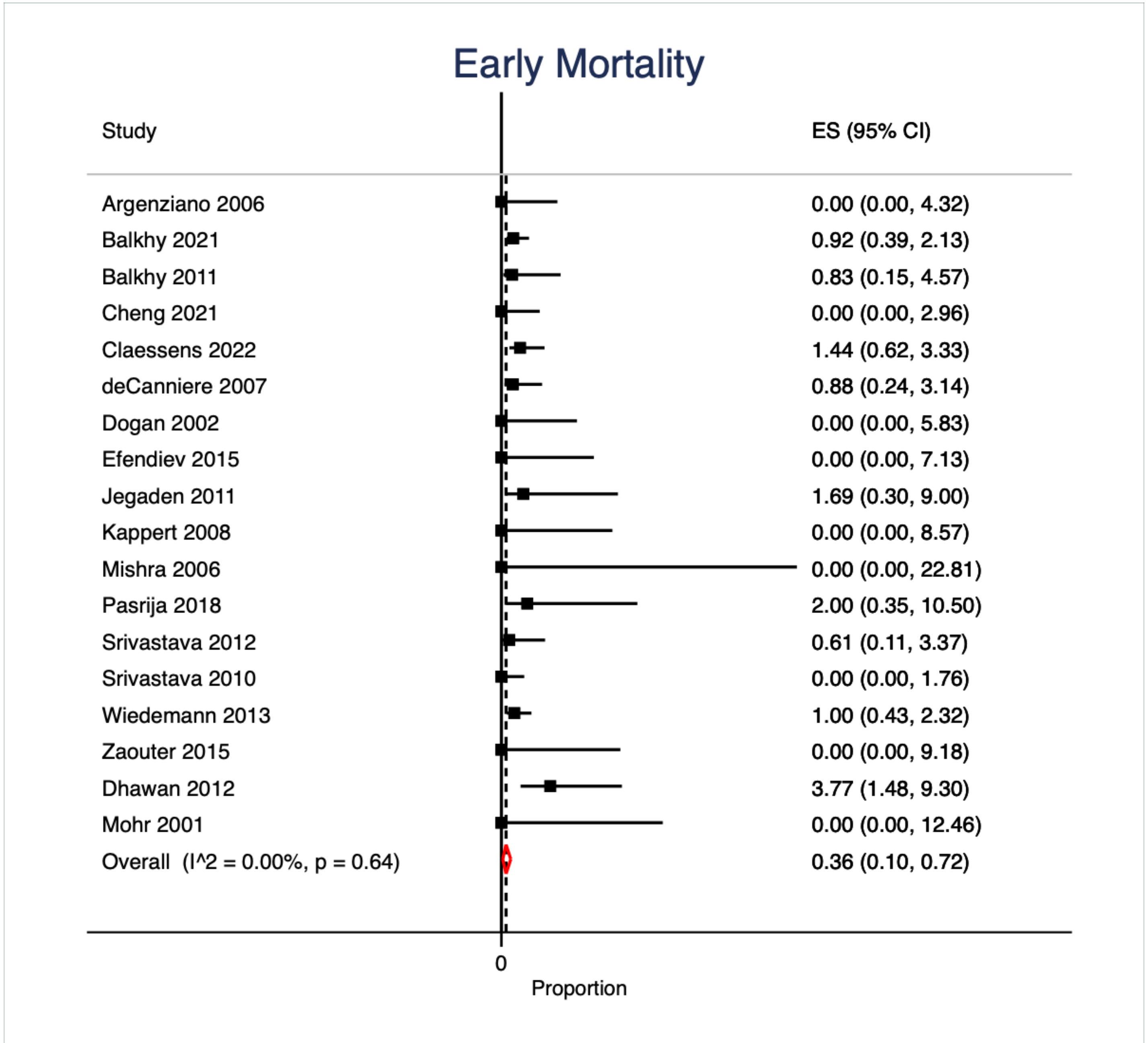

Supplemental Figure 4. Forest plot diagram demonstrating data for late mortality event rates.

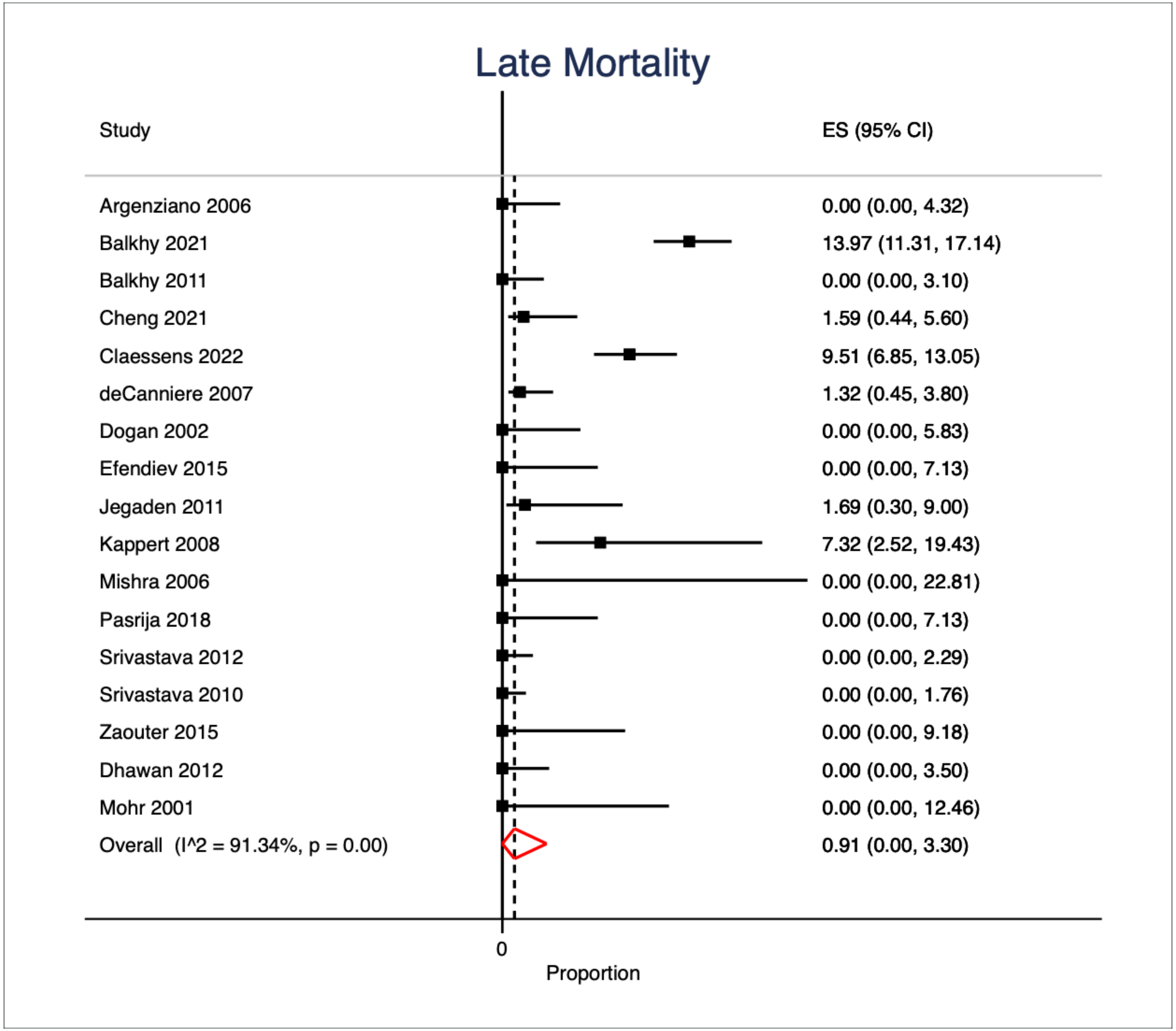

Supplement: sj-pdf-1-inv-10.1177_15569845241296530 – Supplemental material for Totally Endoscopic Coronary Artery Bypass Graft: Systematic Review and Meta-Analysis of Reconstructed Patient-Level Data [file sj-pdf-1-inv-10.1177_15569845241296530.pdf]
